# The Effect of *Otostegia* *persica* in Combination with Chloroquine on Chloroquine-Sensitive and Chloroquine-Resistant Strains of *Plasmodium berghei* Using *In-vivo* Fixed Ratios Method

**Published:** 2012

**Authors:** Mehdi Nateghpour, Leila Farivar, Effat Souri, Homa Hajjaran, Mehdi Mohebali, Afsane Motevalli Haghi

**Affiliations:** a *DepartmentofMedicalParasitologyandMycology, SchoolofPublicHealth, TehranUniversityofMedicalSciences(TUMS), Tehran, Iran. *; b *DepartmentofChemistry, PharmacyFaculty, TehranUniversityofMedicalSciences, Tehran, Iran*

**Keywords:** Otostegia persica, Plasmodium berghei, Drug combination, Fixed ratios, Chloroquine

## Abstract

Malaria is one of the worldwide parasitic diseases which threaten the life of hundreds of millions of people at the malarious areas each year. The emergence of chloroquine-resistant strains of *Plasmodium falciparum *in most of the malarious areas has encountered the relevant countries with some difficulties about treating the acute cases of the disease particulary if the monotherapy regimen has been used. Because of many advantages for the combination therapy, the effectiveness of chloroquine (CQ) and *Otostegia persica *(OP), a medicinal plant in combination form, was tested against the chloroquine-sensitive and chloroquine-resistant strains of *Plasmodium berghei* in sourian mouse using *in-vivo* adapted fixed ratios method in this study.

At the first step, ED_50_s (50% effective dose) of chloroquine and *O. persica* against both CQ-sensitive and CQ-resistant strains of *P. berghei *were calculated using *in-vivo* test in the mice. Ratios of 0, 10, 30, 50, 70, 90 and100% from each ED_50_ were prepared and contrarily combined together to make the following fixed ratios of 0/100, 10/90, 30/70, 50/50, 70/30, 90/10, and 100/0 of CQ/OP and the parasites were exposed to the combined ratios. Determination of ED_50_s showed 1.1 mg/Kg and 2.4 mg/Kg of mouse body weight for chloroquine in CQ-sensitive and CQ-resistant strains respectively and 450 mg/Kg for *O. persica* in both strains. The results also showed that the combinations of “50% CQ + 50% OP”, “30% CQ + 70% O.P” and “70% CQ + 30% OP” were more effective than other combinations against CQ-sensitive strain. The fixed ratio combinations of chloroquine and *O. persica* showed an additive in CQ-resistant strain. Toxicity consideration showed no toxic effect of the combinations on the mice.* Otostegia persica* potentiated the effectiveness of chloroquine against the chloroquine-sensitive strain of *P. berghei* but not on chloroquine-resistant *P. berghei*. Moreover, the greatly modified fixed ratios method in this study can be considered as useful methods for *in-vivo* combination tests in murine malaria parasites.

## Introduction

Malaria, the most important parasitic life threating disease in the malaria-affected areas, is a hematoprotozoan infection caused by four species of *plasmodium*. Although malaria is cured by some effective antimalarial drugs, at present, the medicinal treatment of the infection has encountered with an immense obstacle due to the emergence of drug resistance phenomenon in some species of *plasmodium*. *Plasmodium falciparum* is highly resistant to chloroquine (CQ) in Iran and many of the malarious countries and more or less to a number of other antimalarials in some malaria endemic areas (1-3). Moreover, some reports indicate the emergence of chloroquine-resistant strains of *Plasmodium vivax *in a number of malarious countries (4-7). In view of the increasing incidence of *Plasmodium falciparum *and *Plasmodium vivax *drug-resistant strains besides preparing new antimalarials, effective approaches are also needed that might delay the emergence of the resistance. Combination therapy is a highly recommended pathway for delaying the emergence of drug resistance or potentiating the effectiveness of antimalarials against the resistant strains in Plasmodia (8). Such demand is more emphasized when the combination takes place between medicinal herbal extracts and synthetic antimalarials. *Otostegia persica (OP*) is an endemic medicinal plant growing in south and southeast parts of Iran. The equeous extract of the plant is traditionally used as antispasmodic, anti-arthritis and antipyretic (9) through the local habitants.

*Otostegia persica* has a modest action against the sexual forms of CQ-sensitive *Plasmodium berghei* and is non-toxic to the mouse at antiparasitic doses (10). Despite many uses of *O. persica*, little data can be found about the biological activity of the plant. Producing an experimental CQ-resistant strain of *Plasmodium berghei *derived from CQ-sensitive parasite in our malaria laboratory (11), excited us to consider the effect of *Otostegia persica* in combination with chloroquine against the CQ-resistant and CQ-sensitive strains of *Plasmodium berghei* in sourian mice using fixed ratio method as a first study among its category.

## Experimental

Experiments were conducted on sourian male mice weighing between 20 and 25 g. Animals were kept in plastic and comfortable cages under a normal light-dark cycle, fed on mice pellets and received tap water.

*Parasites*

Chloroquine-sensitive NICD strain of *Plasmodium berghei* stored in liquid nitrogen (originally from Haffkine Institue**,** India) was maintained in mice through a syringe passage, a couple of weeks before the experiments.

*Drugs*

*Otostegia persica *(Labiatea) plant was collected from Hormozgan province, a malarious area in southeast of Iran, and the aerial parts of the plant were powdered for the extraction. Powdered parts were extracted with 96˚ ethanol and the alcohol was evaporated under the pressure of vacuum. The extract was made up as a stabilized suspension to produce concentrations of 100, 200, 350 and 450 mg/Kg. For achieving the stabilized suspension, the extract was dissolved in 2.5% Tween 80 (diluted in normal saline) under the sonicating situation. Chloroquine diphosphate (sigma chemical Co.) was dissolved in distilled water to make up 1, 3, 10, 20 and 30 mg/Kg concentrations.

*Methods*

The cryopreserved parasites were thawed and suspended in physiological saline to be made up a volume of 0.2 mL suspension. A number of mice were infected via the intraperitoneal route and allowed parasitemia to develop up to approximately 10% (by day 4 after the infection). The infected blood was then collected into the heparinized tubes directly from heart via the cardiac puncture under the ether anesthesia. The blood was diluted in physiological saline at the ratio of 10^6^ parasitized erythrocytes in 0.2 mL of the dilution. The prepared suspension was injected subcutaneously in mice. To determine the drug doses for producing 50% suppression of parasitemia for chloroquine and *O. persica*, a 4-day suppressive test was conducted (12). For each previously mentioned concentrations of chloroquine and *O. persica*, five mice were appropriated. *P. berghei*-infected erythrocytes were injected on day 0 and the drugs were given during the days 0 to 3. The first dose was administered 2 h after the injection of the infected erythrocytes on day 0. On the 4^th^ day, the parasitemia of the mice was estimated using Giemsa-stained thin tail-blood smears. For each compound, ED_50_ was calculated from the graphs drawn on semi-log papers against both chloroquine-sensitive and chloroquine-resistant* P. berghei. *Five uninfected and five untreated mice were allocated for each line of the tests as the control groups. The *in-vitro* fixed ratios technique of determining the influence of one drug on the activity of another based on the predetermined inhibitory concentration 50% (IC_50_) values as described by Chawira and Warhurst (13) with considerable modifications, was adapted for *in-vivo* tests in this study as followes. Aliquots of 0.2 mL containing fifty percent of effective dose (ED_50_) concentrations of chloroquine and *O. persica* alone (in the ratios of 90%:10%, 70%:30%, 50%:50%, 30%:70% and 10%:90% respectively) of the first and second drug solutions were injected subcutaneously into the relevant groups of mice. The number of mice in each group and control sets were similar to that described for ED_50_s determination. Percentage inhibition values and standard deviation (SD) for each ratio were calculated. The results of each combination were plotted on a figure containing two vertical axes for percentage inhibition values of the ED_50_ concentrations of the individual drugs and a horizontal axis for fixed ratios of combined concentrations. The points of ED_50_ values on the vertical axes were joined through a straight line. The influence of *O. persica *on the effectiveness of chloroquine against the parasites was shown as a point according to the relevant fixed ratios. When the points take place above the line, it is considered as the potentiation of the activity between *O. persica* and chloroquine against the parasites. When they fall below the line, it is assumed as an antagonistic activity. Points lying on the line indicate an additive effect. The treated mice were reassessed through the thin smears on days 7, 14 and 21 to be noted weather the recrudescence had been occurred. Toxicity of the combination at the combined ratios was assessed against the mice using 3 mice for each fixed ratio of the combination that received a subcutaneous injection daily for 14 days. Subsequently, at the end of the injection period, clinical situations of the mice were followed up until day 50.

**Table 1:** ED_50_ concentration of chloroquine and O.persica against the chloroquine-sensitive and chloroquine-resistant strains of *P.berghei*.

**Parasites**	**Drugs**			
	**Chloroquine**		***O.persica***	
	MeanED_50_ (mg/Kg)	Inhibition% (± SD)	MeanED_50_ (mg/Kg)	Inhibition% (± SD)
Chloroquine	1.1	50.2	450	46.2
-sensitive *P.berghei*		(± 5.3)		(± 2.4)
Chloroquine	24	50	450	42.6
-resistant *P.berghei*		(± 4.7)		(± 3.7)

## Results and Discussion

**Figure 1:**
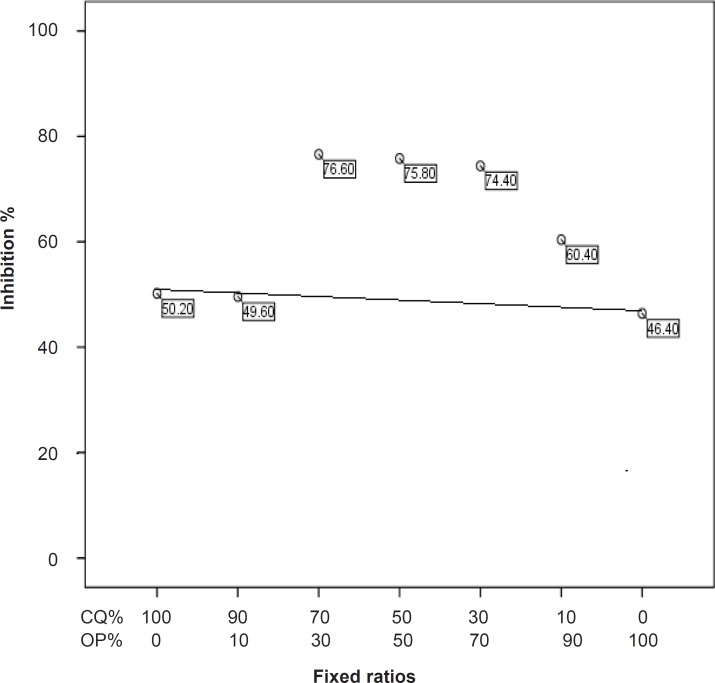
Interaction between chloroquine and *O.persica* on the chloroquine-sensitive strain of *P.berghei.*

**Figure 2:**
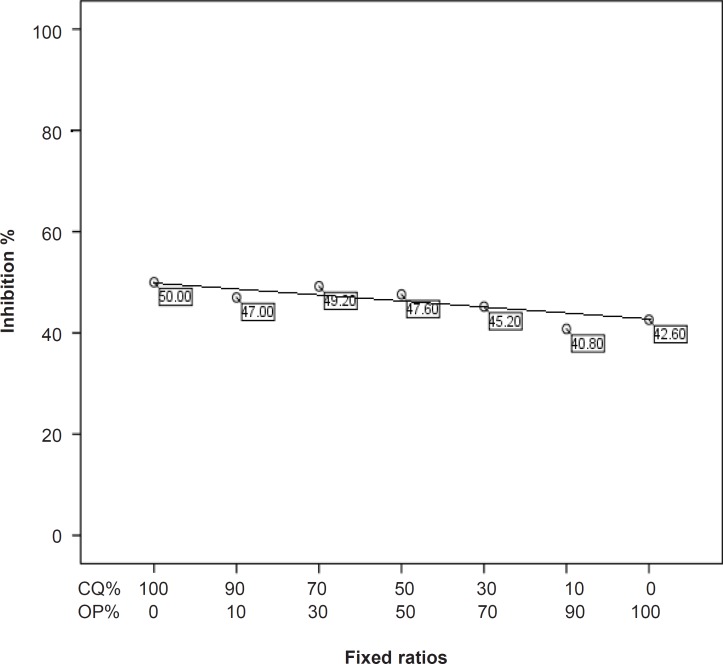
Interaction between chloroquine and *O.persica* on the chloroquine-resistant strain of *P.berghei*

Fifty percent of effective dose values of chloroquine and *O. persica* tested against chloroquine-sensitive and chloroquine-resistant strains of *P. berghei* have been tabulated in Table 1. The results of the interaction between chloroquine and *O. persica* on chloroquine-sensitive and chloroquine-resistant strains of *P. berghei* are graphically shown in Figures 1 and 2 and given in Tables 2 and 3. The combination between chloroquine and *O. persica* demonstrated marked potentiating effects on chloroquine-sensitive strain (NICD strain ) in ratios of 70% CQ + 30% OP, 50% CQ + 50% OP and 30% CQ + 70% OP but not in other ratios (Table 2). Additive effects were seen in combination between chloroquine and* O. persica* against the chloroquine-resistant strain (TUMS/PB/R strain) in 90% CQ + 10% OP, 70% CQ + 30% OP, 50% CQ + 50% OP and 30% CQ + 70% OP ratios, but an antagonism was detected in the ratio of 10% CQ + 90% OP (Table 3). The results showed that the average survival time of the five mice (for each concentration) infected with chloroquine-sensitive parasites after treating with combined doses of 70% CQ + 30% OP, 50% CQ + 50% OP and 30% CQ + 70% OP ratios with 20.4, 21.2 and 21 days respectively, was longer than those treated mice infected with chloroquine-resistant strain (p < 0.05). The result obtained from the toxicity considerations did not reveal any clinical deleterious manifestation until fifty days follow up.* Otostegia persica*, originally an Iranian plant, is usually employed as an antipyretic traditional remedy in malarious south and southeastern areas of Iran. Little data can be found about *O. persica *in the literatures. Some pharmacokinetics, antibacterial , antiparasitic and antiplasmodial descriptions were reported by a number of authors (10, 14- 16). Combination therapy in malaria is considerably recommended both in drug-sensitive and drug-resistant infections particularly in* falciparum* malaria (8, 17). The interaction between chloroquine and *O. persica* against the two strains of *P. berghei*, the murine malaria parasites, was considered in this study.* Plasmodium berghei* is an acceptable animal model for the chloroquine-sensitive and chloroquine-resistant *Plasmodium falciparum* malaria. In fact, although the combination therapy could not interrupt the establishment of drug-resistance in malaria parasites, it could considerably delay the extension of phenomenon (17). Our findings showed that *O. persica* potentiated the effect of chloroquine on the chloroquine-sensitive *P. berghei* whilst an additive effect was observed against the chloroquine-resistant strain of the parasite. The interaction between chloroquine and *O. persica* in the ratio of 30% CQ + 70% OP implied that even lower amount of chloroquine can potentiate the effect of *O. persica* on chloroquine-sensitive *P. berghei*. This may be interpreted that such native herb with low side-effects can fill more portion than chemical compound in combined drugs. Potentiating the effect of chloroquine through *O. persica* in combination form against the chloroquine-sensitive *P. berghei* implies that the drugs may share the similar mechanisms of the action on parasites. On the other hand, some authors obtained additive results between chloroquine and artemisinin, originally a Chinese traditional plant, because of their different modes of action on chloroquine-sensitive *P. berghei* and *P. falciparum *strains (17-19). The additive result of the chloroquine and *O. persica* combination on the chloroquine-resistant *P. berghei* obtained in this study confirms the mentioned hypothesis. In other words, the establishment of resistance to chloroquine in *P. berghei* aborts effective interaction of the plant with chloroquine on the parasite.

**Table 2:** Fixed ratios of chloroquine and *O.persica* combination and their relevant effective doses on the chloroquine-sensitive strain of *P.berghei.*

**Groups**	**Fixed ratios**	**Mean effective doses (** ***X*** ± **SD) **
1	100% CQ	50.2 ± 5.3
2	90% CQ + 1O% OP	49.6 ± 3.5
3	70% CQ + 30% OP	76.6 ± 4.6
4	50% CQ + 50% OP	75.8 ± 5.9
5	30% CQ + 70% OP	74.4 ± 3.7
6	10% CQ + 90%OP	60.4 ± 2.8
7	100% OP	46.4 ± 9.1

**Table 3:** Fixed ratios of chloroquine and O.persica combination and their relevant effective doses on chloroquine-resistant strain of *P.berghei.*

**Groups**	**Fixed ratios**	**Mean effective doses (** ***X*** ± **SD)**
1	100% CQ	50 ± 4.7
2	90% CQ + 1O% OP	47 ± 4.7
3	70% CQ + 30% OP	49.2 ± 8.7
4	50% CQ + 50% OP	47.6 ± 6.8
5	30% CQ + 70% OP	45.2 ± 6.7
6	10% CQ + 90%OP	40.8 ± 8
7	100% OP	42.6 ± 3.7

*Otostegia persica* potentiated the effectiveness of chloroquine against the chloroquine-sensitive strain of *P. berghei* but not on chloroquine-resistant *P. berghei*. Moreover, the greatly modified fixed ratios method in this study can be considered as a useful method for *in-vivo* combination tests in murine malaria parasites.
